# Exosomes from hyperglycemia-stimulated vascular endothelial cells contain versican that regulate calcification/senescence in vascular smooth muscle cells

**DOI:** 10.1186/s13578-018-0263-x

**Published:** 2019-01-03

**Authors:** Shuang Li, Jun-Kun Zhan, Yan-Jiao Wang, Xiao Lin, Jia-Yu Zhong, Yi Wang, Pan Tan, Jie-Yu He, Xing-Jun Cui, Yi-Yin Chen, Wu Huang, You-Shuo Liu

**Affiliations:** Department of Geriatrics, Institute of Aging and Geriatrics, The Second Xiangya Hospital, Central South University, 139 Renmin Road, Changsha, 410011 Hunan People’s Republic of China

**Keywords:** Vascular smooth muscle cells, Calcification, Aging, Exosomes, VCAN, Mitochondria

## Abstract

**Background:**

To determine whether and how exosomes from human umbilical vein endothelial cells (HUVEC-Exos) regulates vascular smooth muscle cells (VSMCs) calcification/senescence in high glucose condition.

**Methods:**

HUVEC-Exos were isolated from normal glucose (NG) and high glucose (HG) stimulated HUVECs (NG/HG-HUVEC-Exos) by super speed centrifugation. HUVEC-Exos were identified by transmission electron microscopy and Western blot of CD63. Protein profile in HUVEC-Exos was examined to screen the candidate molecules that mediate HUVEC-Exos function. VSMCs were incubated with HUVEC-Exos. A series of functional assays in vitro were performed to assess the effects of HUVEC-Exos on the calcification/senescence of VSMCs. The role of the candidate protein in HUVEC-Exos-induced VSMCs dysfunction was assessed.

**Results:**

Exosomes isolated from HG-HUVEC-Exos induced calcification/senescence in VSMCs as assessed by Alizarin Red Staining, senescence-associated β-galactosidase (SA-β-gal) staining, and the expression of ALP and p21. HG-HUVEC-Exos significantly increased LDH activity, as well as the product of lipid peroxidation (MDA content), and decreased oxidative stress marker activity, as compared with NG-HUVEC-Exos. Moreover, mechanism studies showed that mitochondrial membrane potential and the expression levels of mitochondrial function related protein HADHA and Cox-4 were significantly decreased in HG-HUVEC-Exos compared to controls. Proteomic analysis showed that HG-HUVEC-Exos consisted of higher level of versican (VCAN), as compared with NG-HUVEC-Exos. Observation under laser confocal microscopy revealed that most green fluorescence of VCAN could overlap with the red fluorescence came from mitochondria, indicating VCAN is mainly localized to the mitochondria of VSMCs. Knockdown of VCAN with siRNA in HUVECs, inhibited HG-HUVEC-Exos-induced mitochondrial dysfunction and calcification/senescence of VSMCs.

**Conclusions:**

Our data indicate an intracellular role for VCAN in VSMCs. VCAN participates in hyperglycemia-induced calcification/senescence via modulation of mitochondrial function in VSMCs.

**Electronic supplementary material:**

The online version of this article (10.1186/s13578-018-0263-x) contains supplementary material, which is available to authorized users.

## Background

In 2013, it was estimated that 382 million people had diabetes worldwide, and by 2035, this was predicted to rise to 592 million [1]. Diabetes and associated complications give rise to a tremendous burden on the healthcare and present major challenges to patients and national economies. Morbidity and mortality of diabetic patients are substantially aggravated by vascular complications including coronary artery, cerebrovascular, and peripheral artery disease. Vascular calcification/aging could influence the threshold, process, severity and prognosis of diabetic vascular complications [2, 3]. A major determinant of vascular aging is vascular calcification, characterized by vascular smooth muscle cells (VSMCs) calcification (Monckeberg’s calcification). Transdifferentiation of VSMCs into osteoblasts is considered to be the most critical pathophysiological of VSMCs calcification [4–7]. There is accumulating evidence suggesting that VSMCs calcification/senescence have central roles in the development and progression of diabetes-related cardiovascular disorders [4, 8].

The vascular response to hyperglycemia is a multifactorial process involving endothelial cells (ECs) and VSMCs, although the mechanism by which the information in circulating blood are transferred from ECs to VSMCs is yet to be understood. Signaling between ECs and VSMCs is crucial for the pathogenesis of diabetic vascular calcification/aging. However, how does circulating high glucose affect the calcification/senescence of VSMCs that are not directly contact with the blood? Exosomes, small vesicles with a diameter of 40–100 nm released from various cell types, have gained much attention for their role in intercellular communication [9–11]. Exosomes can transfer active proteins, lipids, small molecules and RNAs from their cell of origin to the target cell [9]. ECs have been demonstrated to secrete exosomes [12, 13], and the transfer of signaling molecules by exosomes may thus provide a way for communicating between ECs and VSMCs. Similarly, prior study has demonstrated that exosomes from senescent ECs promotes VSMCs calcification [14].

Oxidative stress plays a pivotal role in the pathogenesis of diabetic vascular disease. Mitochondria are important cellular organelles, as well as major sites of reactive oxygen species (ROS) generation. Hyperglycemia-induced mitochondrial dysfunction promotes ROS accumulation that, in turn, causes cellular damage [15, 16]. Several studies have linked mitochondrial dysfunction to VSMCs calcification/senescence [17, 18]. Cellular senescence shows a series of changes in morphology and physiology, including a flat and enlarged morphology, cell replication stop, changes in aging-related proteins expression, such as p53, p21 and p16 [19, 20]. VSMCs senescence in vitro enhances calcification and osteogenic markers including alkaline phosphatase (ALP), collagen 1, and Runt-related transcription factor (Runx2) expression [6, 7, 10].

Therefore, we proposed that exosomes from human umbilical vein endothelial cells (HUVEC-Exos) release their contents into VSMCs, modulating VSMCs calcification/senescence through regulating mitochondrial function. The aims of the present study were to determine: (1) whether HUVEC-Exos regulate VSMCs calcification/senescence, (2) which contents of such HUVEC-Exos might be involved and the associated mechanism of mitochondrial function.

## Methods

### Cell culture

The HUVECs and VSMCs were obtained from the Type Culture Collection of the Chinese Academy of Sciences (Shanghai, China). The HUVECs were maintained in F12 K (Hyclone, Logan, Utah, USA) with 10% fetal bovine serum (Sigma, St. Louis, MA, USA) and 1% penicillin and streptomycin. The VSMCs were maintained in F12 (Hyclone, Logan, Utah, USA) with 10% fetal bovine serum and 1% penicillin and streptomycin. These cells were maintained under standard cell culture conditions of 37 °C, 5% CO_2_ and 95% humidity.

### Isolation of exosomes from HUVECs

HUVECs were seeded in 55 cm^2^ culture flasks. After incubation in exosomes-free medium with normal glucose (5 mmol/l, NG) or high glucose (30 mmol/l, HG) conditions for 48 h, the cell culture supernatant were collected. The supernatant containing exosomes were centrifuged at 2000*g* for 20 min, 10,000*g* for 30 min and 14,000*g* for 20 min. Then, exosomes were isolated with Exosome Isolation Kit (SBI, Palo Alto, CA, USA) according to the manufacturer’s protocol. All centrifugations were done at 4 °C. Exosomes were stored at − 80 °C or used for the downstream experiments.

### Identification of exosomes

The size and morphology feature of exosomes were examined by transmission electron microscopy as described previously in detail [21]. Briefly, exosomes suspension was mixed with an equal volume of 4% paraformaldehyde and deposited on Formvar-carbon-coated EM grids. Images were acquired with a transmission electron microscope (Hitachi, Tokyo, Japan). Exosomal surface marker protein CD63 was identified by Western blot.

### Calcification assays

VSMCs calcification was assessed by Alizarin Red Staining. Following co-culture with HUVEC-Exos for 10 days, VSMCs were washed twice with PBS and fixed with 4% paraformaldehyde. The cells were exposed to 0.2% Alizarin red (pH 8.3, Solarbio, Beijing, China). Subsequent to washing with PBS, cells were visualized by phase microscopy using an inverted microscope (Olympus Corporation, Tokyo, Japan). The ALP protein expression level was detected using Western blot.

### Senescence analysis

After incubation with HUVEC-Exos for 48 h, VSMCs were fixed in 2% formaldehyde and 0.2% glutaraldehyde for 10 min at room temperature and then washed with PBS. VSMCs senescence was determined with senescence-associated β-galactosidase (SA-β-gal) Staining Kit (Solarbio, Beijing, China) according to the manufacturer’s protocol. The p21 protein expression level was assayed by Western blot.

### Mitochondrial membrane potential assay

The mitochondrial membrane potential was assessed by flow cytometry detection of JC-1 fluorescence (Sigma, St. Louis, MO, USA). After culturing with HUVEC-Exos for 48 h, VSMCs (5 × 10^5^) were harvested by centrifugation (5 min at 500*g*) and then resuspended in 200 μl of RPMI medium without fetal bovine serum. According to the manufacturer’s protocols, cells were left for 20 min at 37 °C in a humidified atmosphere. After this incubation, JC-1 was added to a final concentration of 2.5 μmol/l, and cells were shaken in the dark at 37 °C for 15 min. Afterwards, cells were counted in a BD FACS Calibur (BD Bioscience, San Jose, CA, USA).

### SOD, LDH and MDA determination

As indicators of cellular damage, malondialdehyde (MDA) content and lactate dehydrogenase (LDH) activity were determined using commercial kits (Beyotime, Beijing, China) according to the manufacturer’s instructions. The activity of oxidative stress indicator (superoxide dismutase, SOD) was detected using commercial kit (Beyotime, Beijing, China) according to the manufacturer’s instructions.

### Exosomes labeling and uptake

Exosomes were labeled with the red fluorescent dye CellTracker DiD (AAT Bioquest, Sunnyvale, CA, USA) as described in previous study [22]. Exosomes labeling with CellTracker DiD were performed following the manufacturer’s procedures. Exosomes from 1.5 × 10^8^ HUVECs were resuspended in 200 μl PBS with 12 μl/ml diluted CellTracker DiD. After 20 min of incubation at room temperature, VSMCs were incubated with the CellTracker DiD-labeled HUVEC-Exos at 37 °C for 2 h. VSMCs were then washed with PBS and fixed with 4% paraformaldehyde for 20 min. After washing with PBS, nuclei were stained with DAPI (Invitrogen, Carlsbad, CA, USA). The signals were analyzed with a fluorescence microscope.

### Proteomic analysis of HUVEC-Exos

The HUVEC-Exos samples were processed for iTRAQ-based quantitative proteomic analysis by Jingjie PTM BioLab (Hangzhou, China). We compared the proteomic content of HUVEC-Exos in HG with NG conditions, using high-performance liquid chromatography tandem mass spectrometry (HPLC–MS/MS). Gene Ontology (GO) analysis was performed to classify all identified proteins into three categories (cell component, molecular function and biological process) using the UniprotKB database (http://www.uniprot.org/), InterProScan (http://www.ebi.ac.uk/interpro/) and GO annotation (http://geneontology.org/). Differentially expressed proteins were identified with a cutoff of absolute fold change ≥ 1.3. For each category, a two-tailed Fisher’s exact test was employed to test the enrichment of the differentially expressed protein against all identified proteins. The GO with a corrected p value < 0.05 was considered significant.

### Immunofluorescent staining

VSMCs and HG-HUVEC-Exos were co-cultured in glass coverslips that had been placed in 6-well culture dishes for 48 h, and then were washed with PBS prior to fixation with 4% formaldehyde. After fixation, cells were washed with PBS and then blocked for 20 min with goat serum. Mitochondria were labeled with fluorescent mitochondrial indicator (ATT, Sunnyvale, CA, USA) according to the manufacturer’s protocol. The cells were washed with PBS and incubated overnight at 4 °C with the VCAN antibody (R&D, Minneapolis, Minnesota, USA) at a concentration of 1:200. Cells were then washed with PBS and incubated with Alexa Fluor goat anti-rabbit IgG at a concentration of 1:100 for 40 min at 37 °C. After that, cell nuclei were stained with DAPI. Images were captured using a Leica TSC-SP5 laser confocal scanning microscope.

### Western blot analysis

Cells were lysed in a buffer containing 50 mmol/l TrisHCl, 150 mmol/l NaCl, 10 mmol/l EDTA, 1% Triton X-100, 0.2% NaN_3_, 10 μg/ml Aprotini and protease inhibitors. The lysates were centrifuged at 10,000*g* for 5 min and supernatants were collected. Protein concentrations were determined, and equal amounts of protein were submitted to SDS-PAGE and transferred onto 0.2 μm PVDF membranes (Millipore, Temecula, CA, USA). After transfer to PVDF membranes, the membranes were incubated with antibodies that recognize proteins, such as p21 (Cat. No. 60214-1-Ig, 1:1000 dilution), hydroxyacyl-CoA dehydrogenase/3-ketoacyl-CoA thiolase/enoyl-CoA hydratase, alpha subunit (HADHA) (Cat. No. 60250-1-Ig, 1:500) (Proteintech, Rosemont, IL, USA); CD63 (Cat. No. ab59479, 1:500 dilution), ALP (Cat. No. ab67228, 1:500 dilution), VCAN (Cat. No. ab19345, 1:500 dilution), cytochrome oxidase-4 (Cox-4) (Cat. No. ab110261, 1:1000 dilution) and GAPDH (Cat. No. ab125247, 1:4000 dilution) (Abcam, Cambridge, MA, USA) at 4 °C overnight. The membranes were incubated with horseradish peroxidase-conjugated goat anti-mouse secondary antibody (Cat. No. A32727, Thermo Fisher, Waltham, MA, USA) for 1 h at room temperature. The reaction was visualized with chemiluminescence.

### Knockdown of VCAN with siRNA

The specific small interfering RNA (siRNA) and negative control siRNA were synthesized and purchased from Gene Pharmagps (Shanghai, China). The knockdown of the versican (VCAN) gene was performed using siRNA with the following target sequences: VCAN sense, 5′-GAGGCUGGAACUGUUAUUATT-3′; VCAN antisense, 5′-UAAUAACAGUUCCAGCCUCTT-3′; negative control sense, 5′-UUCUCCGAACGUGUCACGUTT-3′; negative control antisense, 5′-ACGUGACACGUUCGGAGAATT-3′. The HUVECs were seeded in six-well plates at 70% confluence. Either VCAN siRNA or negative control siRNA (2 μmol/l) was added to the cells, which underwent transfection using Lipofectamine 3000 Kit (Thermo Fisher, Waltham, MA, USA) according to the manufacturer’s protocol. Following transfection for 6 h, the cells were exposed to HG condition.

### Real-time PCR analysis

Total RNA was extracted from cells using TRIzol reagent (Invitrogen, Carlsbad, CA, USA). The reverse transcription reaction was performed using 1 μg of RNA and a RevertAid™ H Minus First Strand cDNA Synthesis Kit (Fermentas, Burlington, Ontario, CA), according to the manufacturer’s protocol. For real-time PCR amplification, the cDNA were amplified using SYBR GreenPCR Master Mix (ABI, New York, NY, USA) and 0.4 μmol/l of each primer pair. Amplification was undertaken using an ABI 7900 real-time PCR system (ABI, New York, NY, USA). Quantitation of the data was performed via the 2^−ΔΔCT^ method, using GAPDH gene expression as an endogenous reference. The primer sequences used for the real-time PCR analysis were as follows: VCAN forward, 5′-GTAACCCATGCGCTACATAAAGT-3′; VCAN reverse, 5′-GGCAAAGTAGGCATCGTTGAAA-3′; GAPDH forward, 5′-GGAGCGAGATCCCTCCAAAAT-3′; GAPDH reverse, 5′-GGCTGTTGTCATACTTCTCATGG-3′.

### Statistical analysis

Results were presented as mean ± SEM, and analysis was performed with Statistical Product and Service Solutions (version 13.0). Differences between groups were evaluated by one-way analysis of variance (ANOVA), followed by the Bonferroni post hoc test to assess the significance of differences between two groups. The data were based on three independent experiments. A level of *p *< 0.05 was considered significant.

## Results

### Exosomes extraction and identification

**Fig. 1:**
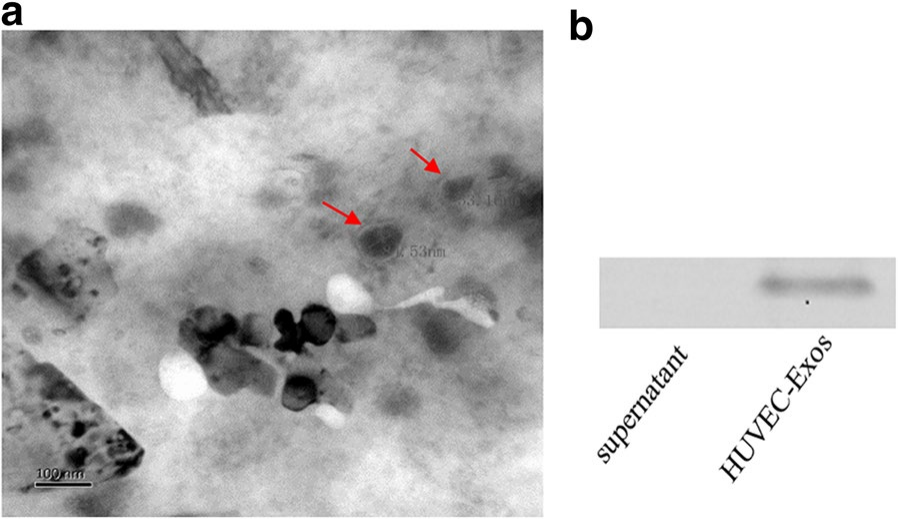
Identification of HUVEC-Exos. The HUVECs were cultured for 48 h in NG (5 mmol/l) or HG (30 mmol/l) conditions. **a** Electron microscopic image of HUVEC-Exos. **b** Western blot of exosomal-specific marker CD63 in HUVEC-Exos. Scale bar, 100 nm

We hypothesized that exosomes were involved in the communication of HUVECs and VSMCs. To test this hypothesis, we first isolated exosomes from the supernatants of NG (5 mmol/l) and HG (30 mmol/l) induced HUVECs. Analysis of transmission electron microscopy showed that the diameter of the HUVEC-Exos were 74.6 ± 8.3 nm (mean ± SEM) (Fig. 1a). Western blot also confirmed the expression of exosomal-specific marker CD63 on the exosome surface (Fig. 1b).

### HG-HUVEC-Exos induce VSMCs calcification/senescence

**Fig. 2:**
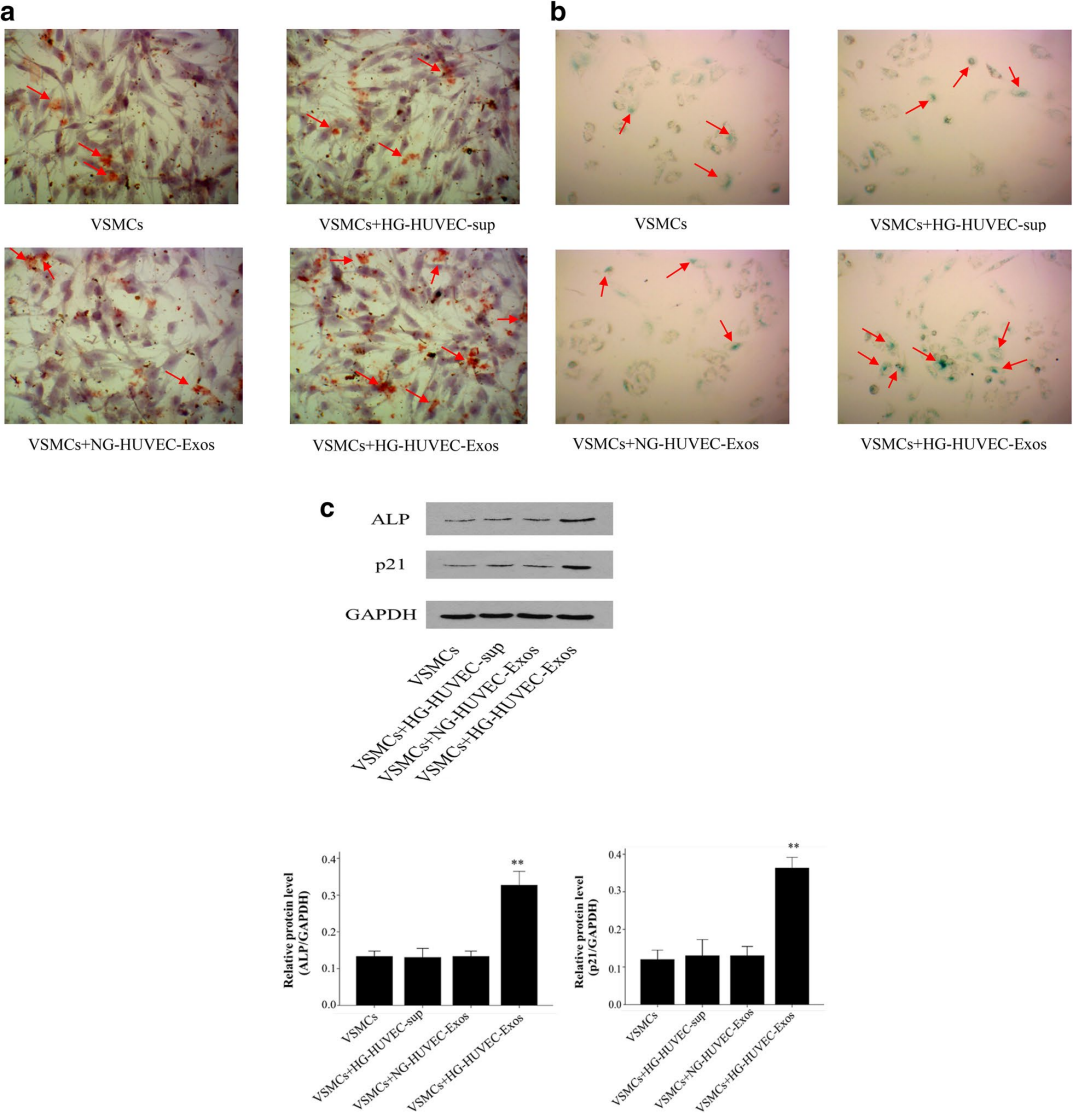
The effects of HUVEC-Exos on VSMCs senescence/calcification. **a** VSMCs were co-cultured with NG/HG-HUVEC-Exos or supernatant isolated from HG stimulated HUVECs (HG-HUVEC-sup) for 10 days, calcification was measured by Alizarin Red Staining (400×). **b** VSMCs were co-cultured with NG/HG-HUVEC-Exos or HG-HUVEC-sup for 48 h, senescence was examined via SA-β-gal staining (400×). **c** The protein expression levels of ALP and p21 were assayed by Western blot. The ALP and p21 levels were quantified and normalised to GAPDH. Data are presented as the means ± SEM of three independent experiments. Statistical analysis was performed using one-way ANOVA test. **p < 0.01 compared with VSMCs group, HG-HUVEC-sup group and NG-HUVEC-Exos group

To determine the ability of HUVEC-Exos in regulating calcification/senescence in hyperglycaemic condition, VSMCs were cultured in the presence of HUVEC-Exos. We found that compared with NG-HUVEC-Exos, HG-HUVEC-Exos induced calcification/senescence of VSMCs as determined by Alizarin Red Staining and SA-β-gal staining, respectively (Fig. 2a, b). These findings were further supported by increased protein levels of ALP and p21 (*p* < 0.01, Fig. 2c), again suggesting that HG-HUVEC-Exos could promote VSMCs calcification/senescence.

### HG-HUVEC-Exos induce mitochondrial dysfunction in VSMCs

**Fig. 3:**
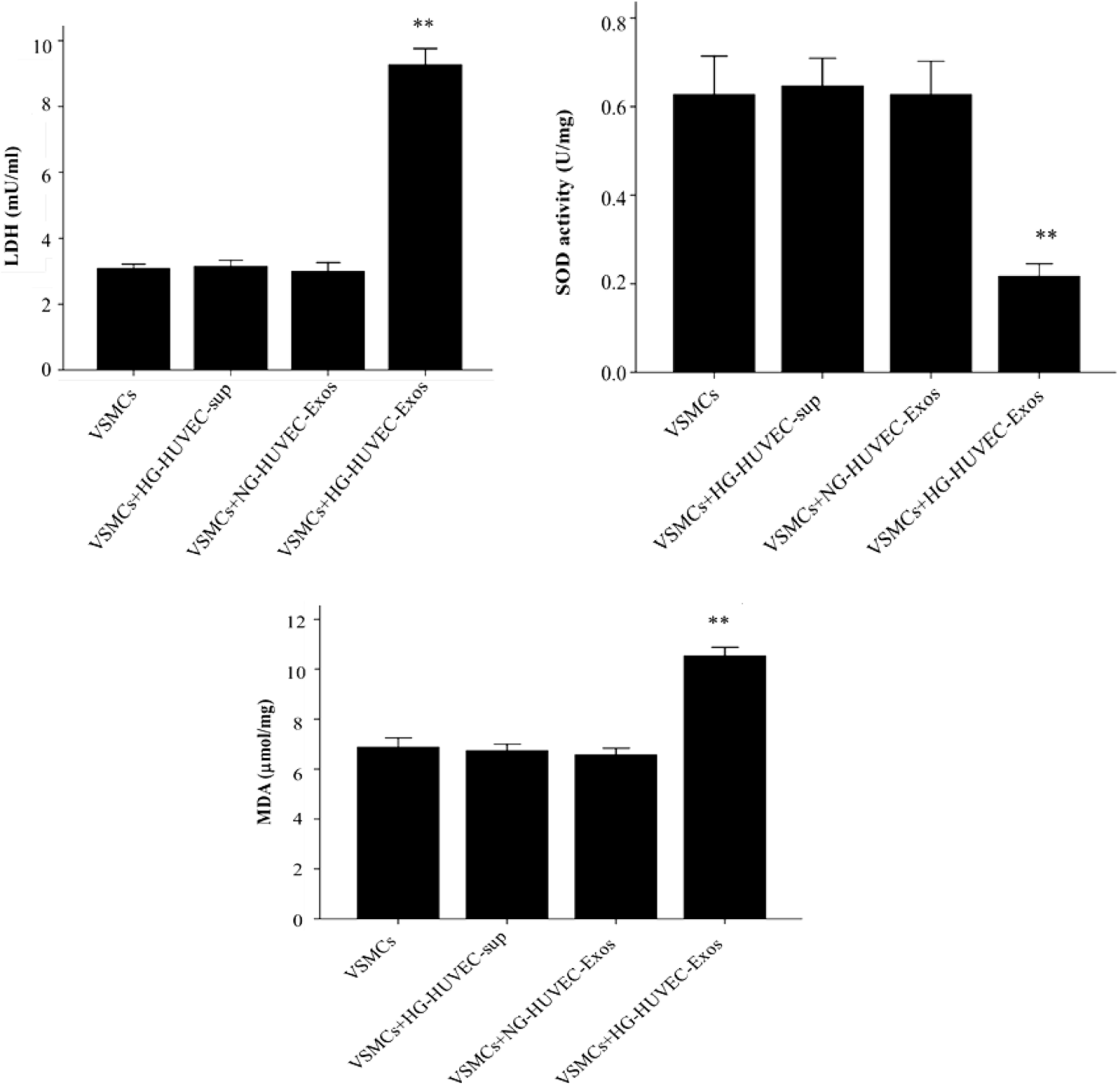
The effects of HUVEC-Exos on MDA content, LDH and SOD activity. VSMCs were co-cultured with HUVEC-Exos or HG-HUVEC-sup for 48 h. MDA content, LDH and SOD activity were determined by using commercial kits. Data are presented as the means ± SEM of three independent experiments. Statistical analysis was performed by using one-way ANOVA test. **p < 0.01 compared with VSMCs group, HG-HUVEC-sup group and NG-HUVEC-Exos group

**Fig. 4:**
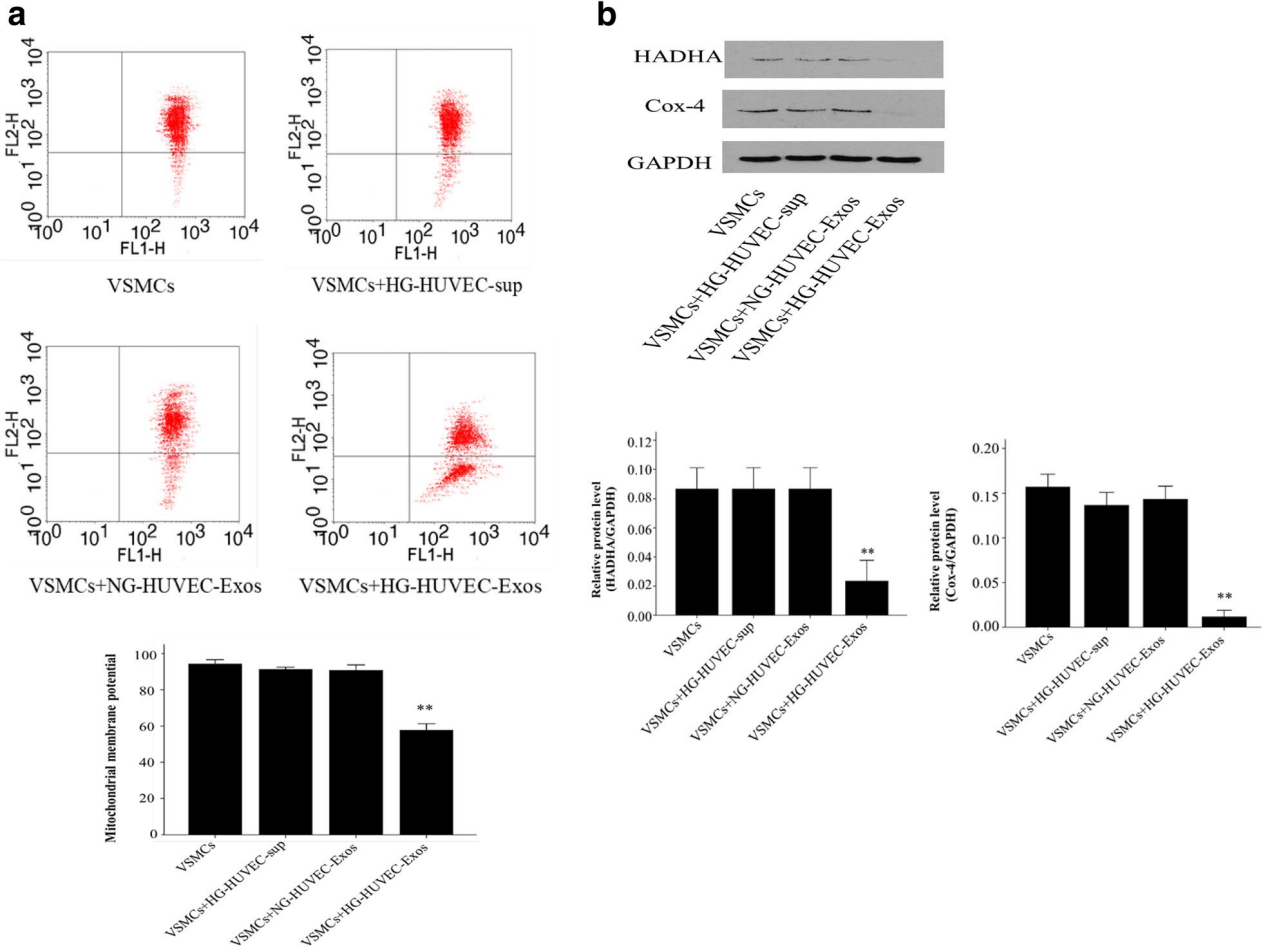
The effects of HUVEC-Exos on mitochondrial function. VSMCs were cultured with HUVEC-Exos for 48 h. **a** Mitochondrial membrane potential was detected by JC-1 staining. **b** The HADHA and Cox-4 levels were analysed by Western blot. The HADHA and Cox-4 levels were quantified and normalised to GAPDH. Data are presented as the means ± SEM of three independent experiments. Statistical analysis was performed by using one-way ANOVA test. **p < 0.01 compared with VSMCs group, HG-HUVEC-sup group and NG-HUVEC-Exos group

Oxidative stress is associated with the pathogenesis of vascular calcification/senescence. We next examined the levels of oxidative stress and cellular damage indicators (LDH, MDA and SOD). As shown in Fig. 3, VSMCs incubated with HG-HUVEC-Exos was associated with a significant up-regulation of LDH activity (*p* < 0.01) and MDA content (*p* < 0.01) and down-regulation of SOD activity (*p* < 0.01) when compared with NG-HUVEC-Exos group. Mitochondria are a major source of ROS. To determine whether mitochondrial dysfunction mediates HG-HUVEC-Exos induced cellular oxidative stress, we examined mitochondrial membrane potential and mitochondrial function related protein HADHA and Cox-4 expression levels. Compared to NG-HUVEC-Exos group, HG-HUVEC-Exos significantly decreased the mitochondrial membrane potential (*p* < 0.01, Fig. 4a) and protein levels of HADHA and Cox-4 (*p* < 0.01, Fig. 4b).

### Capture of HUVEC-Exos by VSMCs

**Fig. 5:**
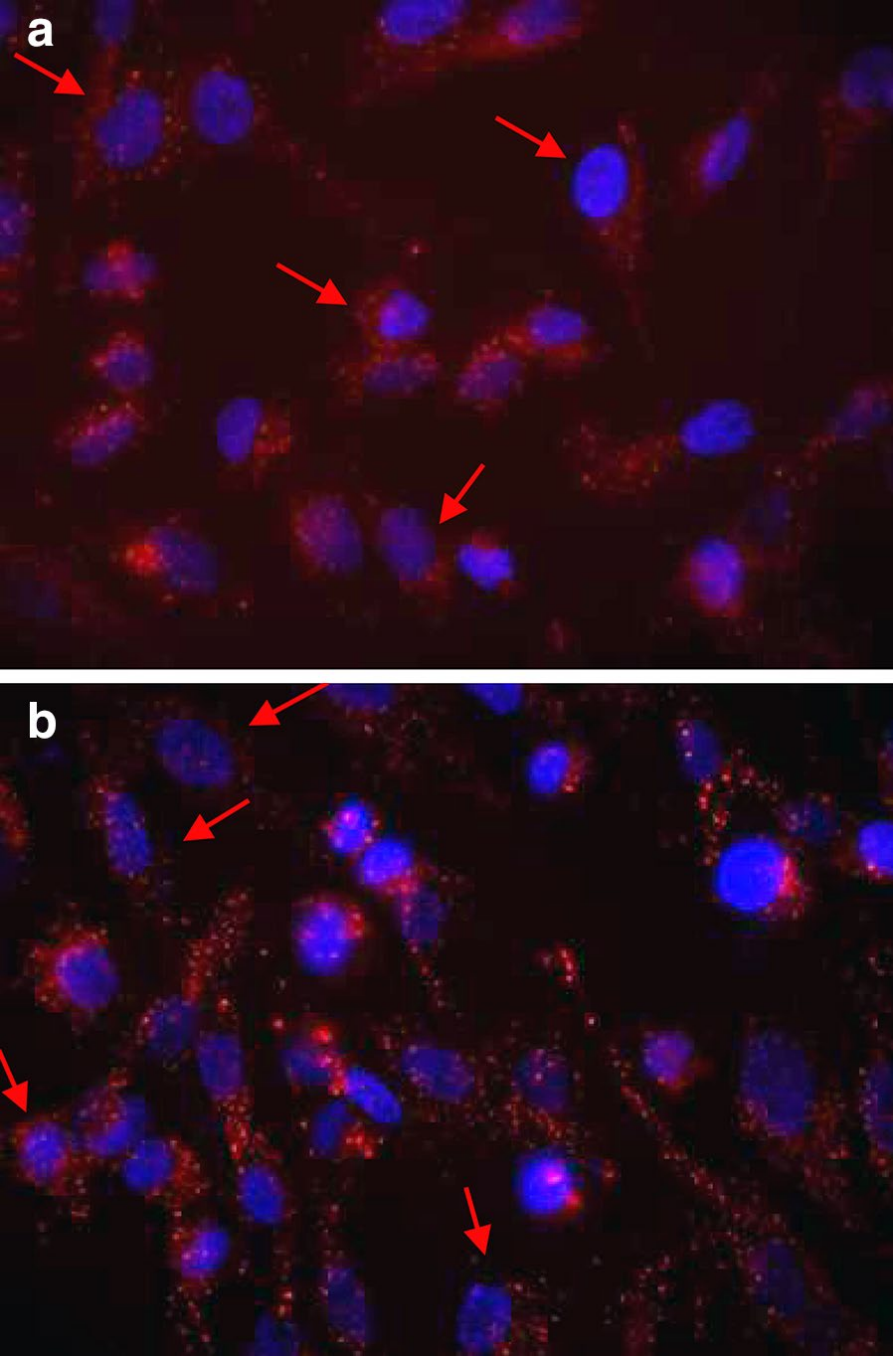
Capture of exosomes by VSMCs. Cells were incubated for 8 h with **a** NG-HUVEC-Exos or **b** HG-HUVEC-Exos stained with CellTracker DiD (red). Nuclei were stained with DAPI (blue). Fluorescence was evaluated by confocal microscopy (400×). A representative experiment from three different experiments is shown

HUVEC-Exos were labeled with CellTracker DiD (red) and cultured for 8 h with VSMCs to determinate whether they regulate calcification/senescence by adherence to cell surface and/or by being endocytosed by VSMCs. Confocal microscopy showed the majority of the HUVEC-Exos were located inside the cytoplasmic compartment of VSMCs (Fig. 5).

### HUVEC-Exos carry proteins

**Fig. 6:**
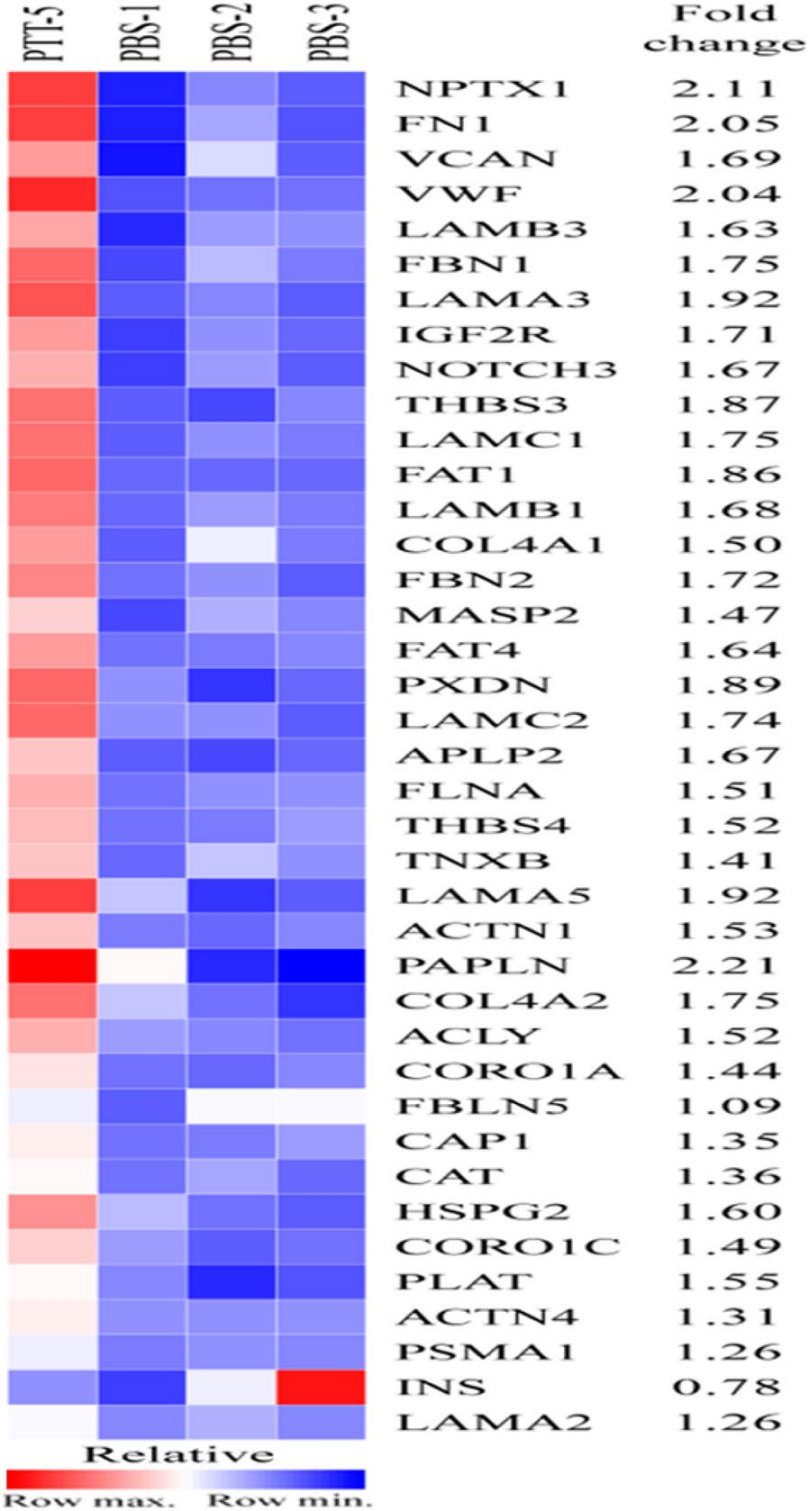
Identification of proteins which were differentially expressed in exosomes. UniprotKB analysis of differential protein expression. PBS1, PBS2 and PBS3: three exosomes samples isolated from HUVECs under NG condition; PTT5: one exosome sample isolated from HUVECs under HG condition

HUVEC-Exos are able to modulate VSMCs calcification/senescence. It was therefore decide to investigate how they may impact on VSMCs function. After successfully isolated exosomes and extracted exosomal proteins from NG and HG culture conditions, we conducted HPLC–MS/MS analysis and identified a total of 569 distinct proteins. 74 proteins were up-regulated and 105 were down-regulated using the cutoff value of 1.3-fold change. The identified proteins were investigated to assess their subcellular localization and molecular function by UniprotKB. These results helped us to focus on VCAN protein. We found that, unlike NG condition, HG treatment was associated with a significant up-regulation of VCAN (Fig. 6).

### VCAN localization and its role in HG-HUVEC-Exos-induced VSMCs calcification/senescence

**Fig. 7:**
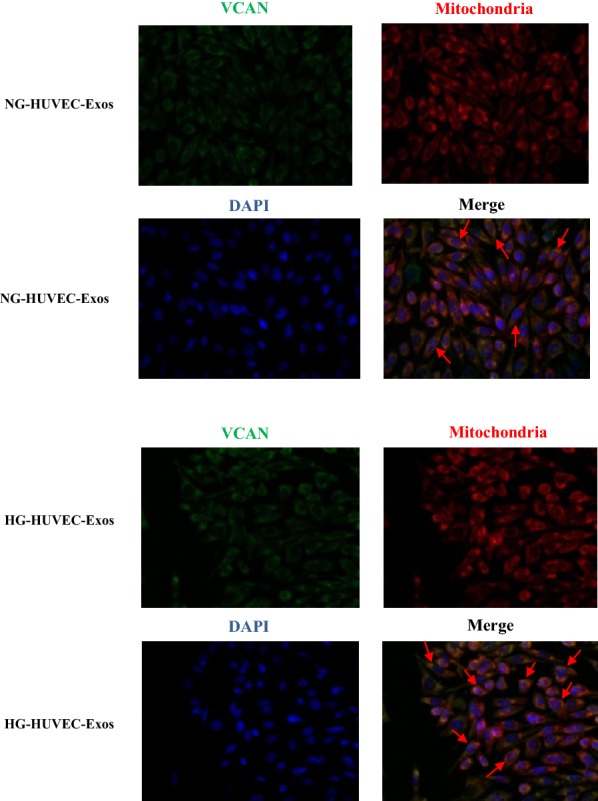
The localization of VCAN in VSMCs. VSMCs were incubated with NG/HG-HUVEC-Exos for 48 h. VCAN (green), mitochondria (red), nucleus (DAPI, blue) (400×). Overlay image showed aggregation of VCAN in mitochondria (orange)

**Fig. 8:**
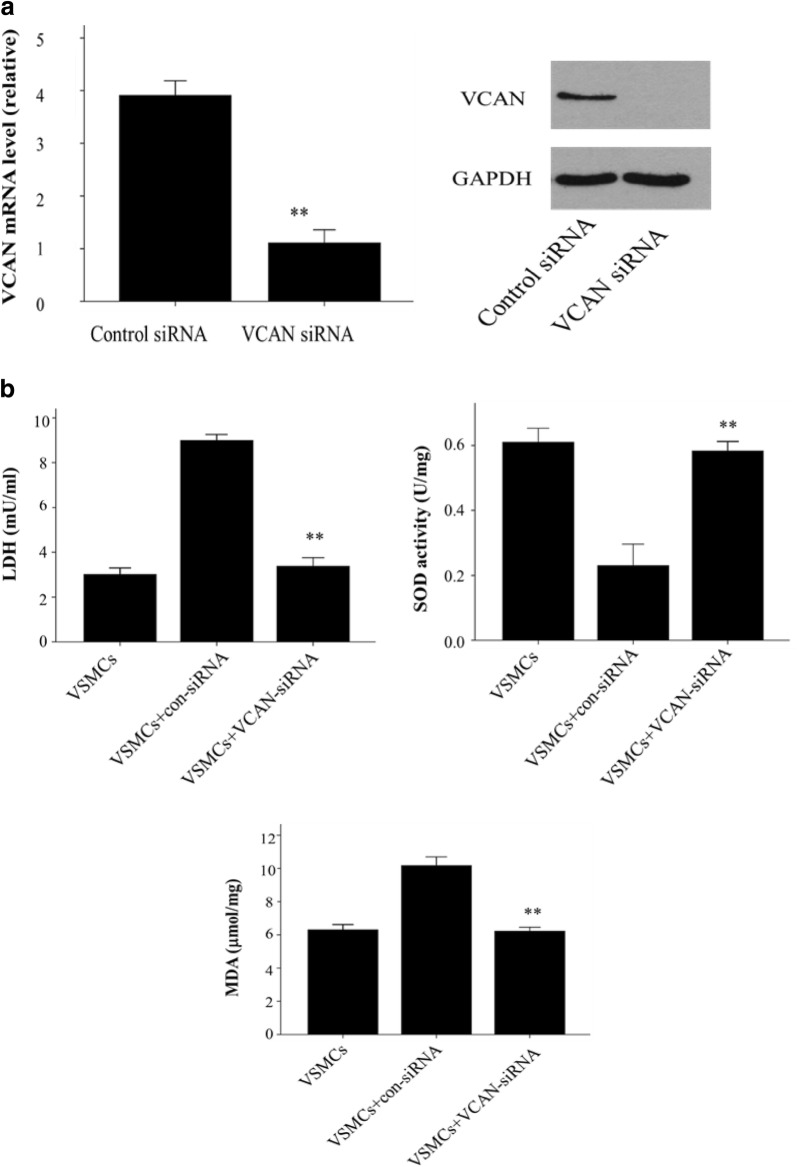
The role of VCAN in HG-HUVEC-Exos-induced VSMCs senescence/calcification. HUVECs were transfected with negative control siRNA (con-siRNA) or VCAN siRNA (VCAN-siRNA) for 6 h and then exposed to HG condition for 48 h. After that, exosomes were isolated and co-cultured with VSMC for 48 h. **a** VCAN mRNA and protein expression were assessed by qRT-PCR and Western blot. **b** MDA content, LDH and SOD activity were determined by using commercial kits. Data are presented as the means ± SEM of three independent experiments. Statistical analysis was performed by using one-way ANOVA test. **p < 0.01 compared with con-siRNA group

**Fig. 9:**
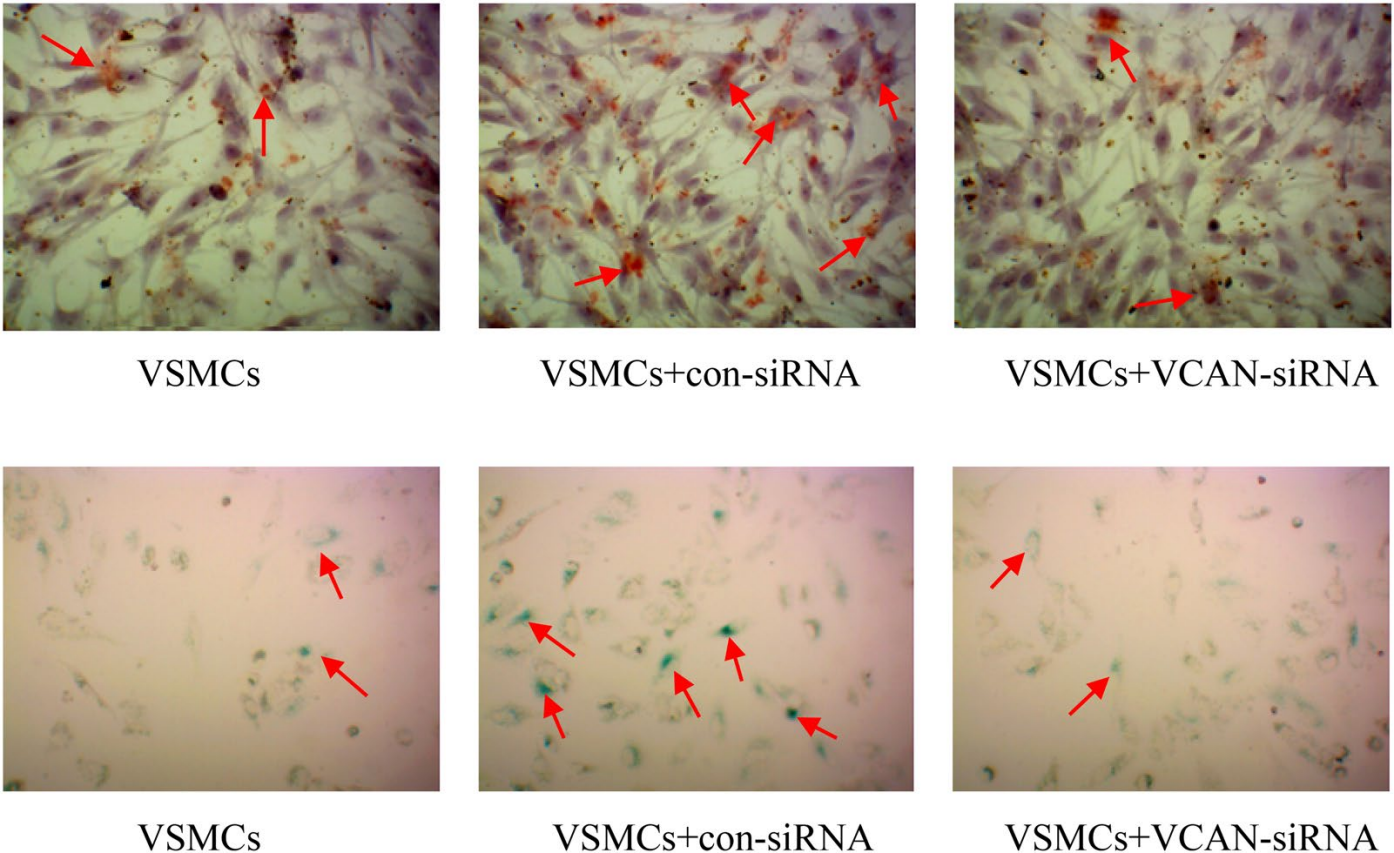
The role of VCAN in HG-HUVEC-Exos-induced VSMCs senescence/calcification. HUVECs were transfected with negative control siRNA (con-siRNA) or VCAN siRNA (VCAN-siRNA) for 6 h and then exposed to HG condition for 48 h. After that, exosomes were isolated and co-cultured with VSMC for 48 h. VSMCs were co-cultured with HUVEC-Exos for 10 days/48 h, calcification/senescence were measured by Alizarin Red Staining/SA-β-gal staining, respectively (400×)

**Fig. 10:**
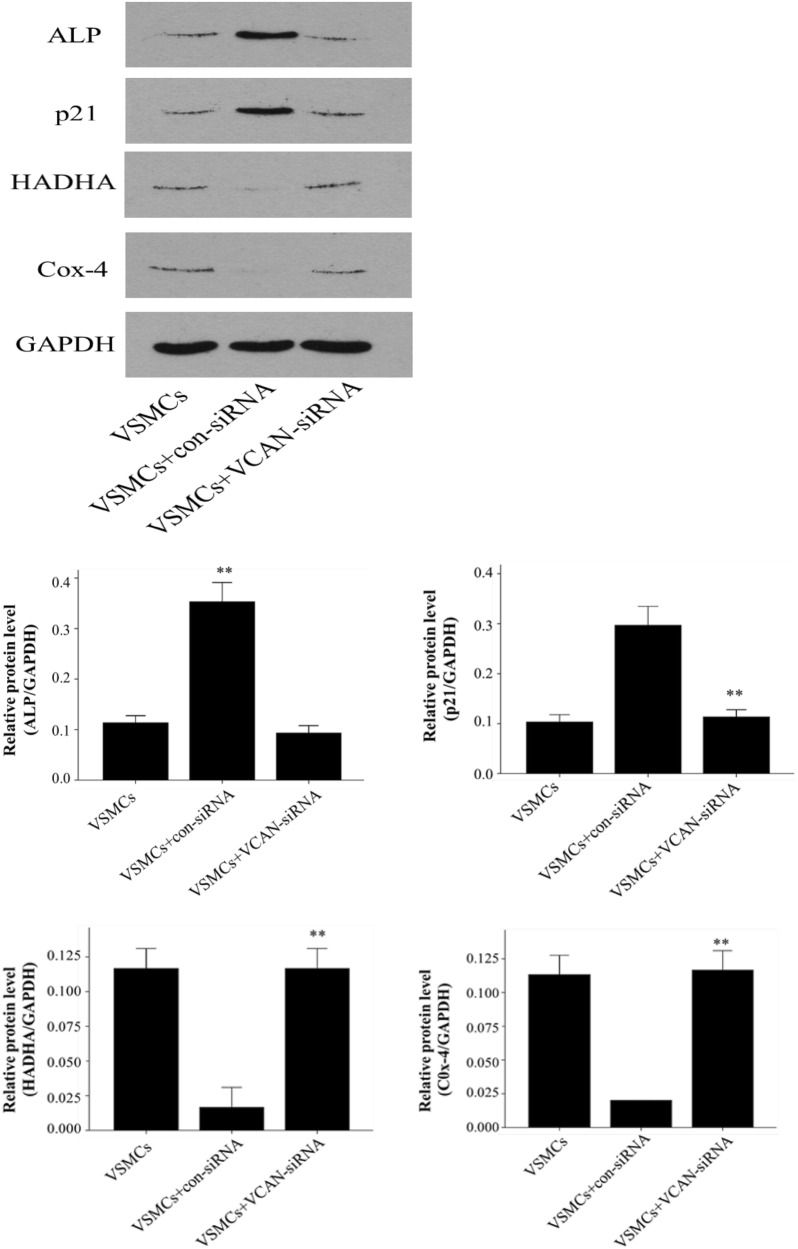
The role of VCAN in HG-HUVEC-Exos-induced VSMCs senescence/calcification. HUVECs were transfected with negative control siRNA (con-siRNA) or VCAN siRNA (VCAN-siRNA) for 6 h and then exposed to HG condition for 48 h. After that, exosomes were isolated and co-cultured with VSMC for 48 h. The protein expression of ALP, p21, HADHA and Cox-4 were examined by Western blot. The ALP, p21, HADHA and Cox-4 levels were quantified and normalized to GAPDH. Data are presented as the means ± SEM of three independent experiments. Statistical analysis was performed by using one-way ANOVA test. **p < 0.01 compared with con-siRNA group

**Fig. 11:**
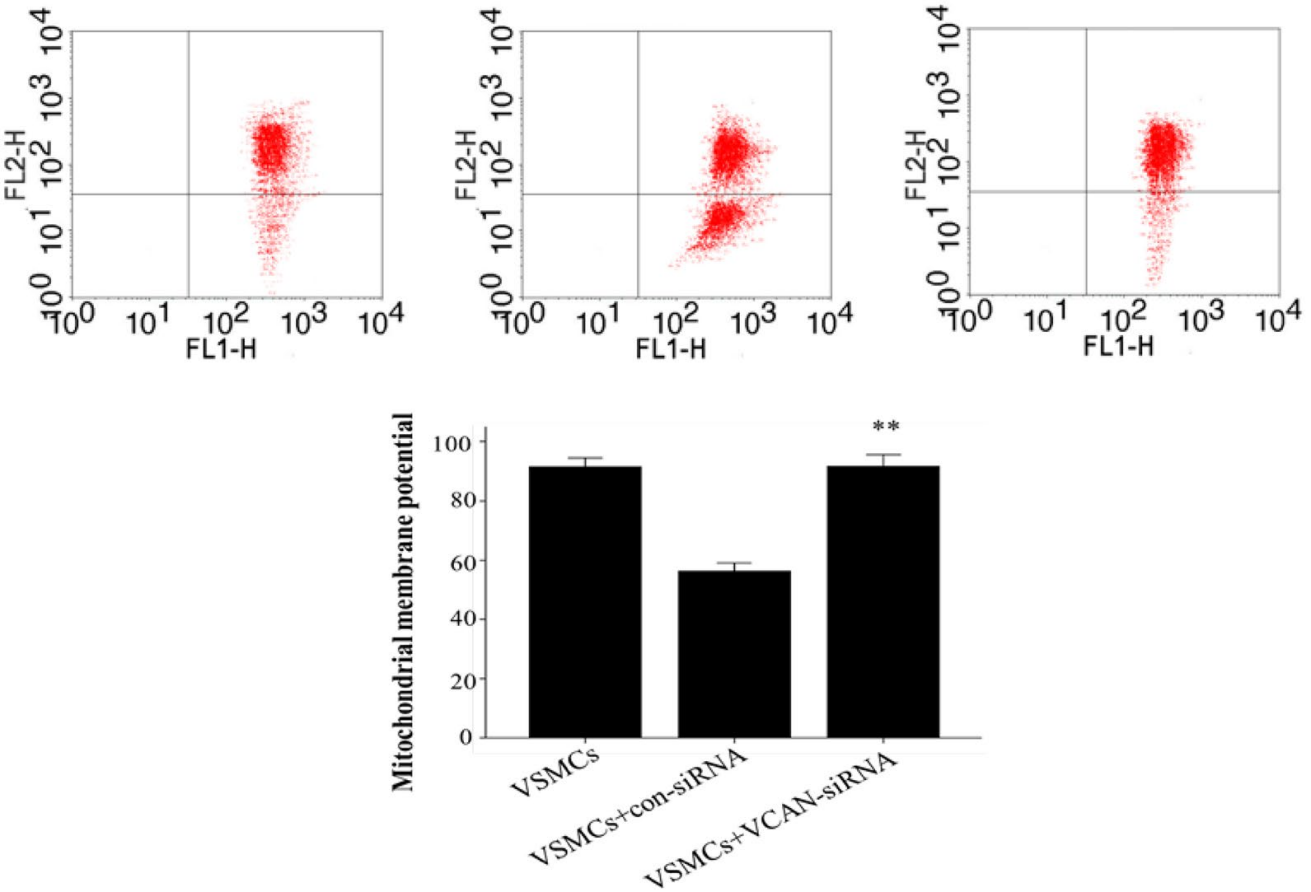
The role of VCAN in HG-HUVEC-Exos-induced mitochondrial dysfunction. HUVECs were transfected with negative control siRNA (con-siRNA) or VCAN siRNA (VCAN-siRNA) for 6 h and then exposed to HG condition for 48 h. After that, exosomes were isolated and co-cultured with VSMC for 48 h. Mitochondrial membrane potential was detected by JC-1staining. Data are presented as the means ± SEM of three independent experiments. Statistical analysis was performed by using one-way ANOVA test. **p < 0.01 compared with con-siRNA group

We have observed that HG-HUVEC-Exos induce mitochondrial dysfunction in VSMCs; thus, we sought to investigate the localization of VCAN in VSMCs. Merged images showed the aggregation of VCAN in the mitochondria (orange), hinting of a mitochondrial localization of VCAN in VSMCs (Fig. 7). To determine the role of VCAN in HG-HUVEC-Exos-induced VSMCs calcification/senescence, VCAN RNA silencing was utilized. As depicted in Fig. 8a and Additional file 1: Figure S1, both VCAN mRNA and protein expression were successfully knocked out by VCAN-siRNA in HUVECs (*p* < 0.01). As expected, HG-HUVEC-Exos with VCAN-depleted were no longer able to either decrease SOD, or increase LDH, MDA levels (*p* < 0.01, Fig. 8b) or induce cellular calcification/senescence in VSMCs (Figs. 9, 10). Knockdown of VCAN upregulated HADHA and Cox-4 protein expression (Fig. 10). Moreover, VCAN knockdown led to upregulation of the mitochondrial membrane potential (Fig. 11). These results suggest that VCAN might be involved in HG-HUVEC-Exos-induced VSMCs calcification/senescence.

## Discussion

Vascular complications account for a substantial proportion of the morbidity and mortality that occurs in patients with diabetes. Vascular calcification/aging are established as severe risk factors for diabetic vascular complications [2, 3]. As a main constituent cell type in tunica-media layer, VSMCs calcification/senescence could be a focal point to explain the pathological progression of diabetic vascular dysfunction. However, how the information in circulating blood is transferred from tunica intima to tunica media? Many studies have found that exosomes can transfer functional molecules to target cells and serve as mediators of cell-to-cell crosstalk under physiological and pathological conditions. Recently, new evidence has revealed that microvesicles produced by senescent cultured ECs promoted calcification in VSMCs [14]. However, in HG environment, whether exosomes can mediate the interaction between HUVECs and VSMCs and participate in the pathogenesis of diabetic vascular damage remains unclear. Our research reveals that VCAN-rich exosomes were secreted from HG-HUVECs and taken up by recipient VSMCs. Upon uptake by VSMCs, VCAN promoted calcification/senescence of VSMCs by inducing mitochondrial dysfunction.

In diabetic patients, long-term hyperglycaemia can induce vascular medial calcification/aging [23]. Cellular senescence is characterized by changes in cell morphology and gene expression. During the onset of vascular calcification, VSMCs in the tunica media transdifferentiate into osteoblast-like phenotype [24]. In accordance with previous study [23], our data suggest that HG-HUVEC-Exos induce VSMCs calcification/senescence, as determined by Alizarin Red Staining and SA-β-gal staining. Furthermore, the protein expression of ALP and p21 in VSMCs were also increased in HG-HUVEC-Exos group than that in NG-HUVEC-Exos group.

Excessive ROS is known to mediate VSMCs calcification/senescence [25–27]. Mitochondria are the primary sources of ROS [28]. In fact, it has been proposed that overproduction of ROS that occurs in response to mitochondrial dysfunction in diabetes, is the major mechanism by which hyperglycemia exerts its influence on vascular damage [29–31]. In this regard, we examined mitochondrial function in VSMCs treated with HUVEC-Exos. Our study showed that elevated glucose concentration results in decreased mitochondrial membrane potential in VSMCs. In addition, HG-HUVEC-Exos decreased the expression levels of Cox-4 and HADHA in VSMCs by 15- and 9-fold compared to NG condition, respectively. Cox is a terminal enzyme of the mitochondrial electron transfer chain. HADHA is the alpha subunit of mitochondrial trifunctional protein. These results above suggest that HG-HUVEC-Exos could induce mitochondrial dysfunction in VSMCs. Our results is consistent with previous studies showing that mitochondrial dysfunction leads to cellular calcification/senescence of VSMCs [25, 32]. MDA, which is produced by lipid peroxidation of polyunsaturated fatty acids, is commonly used for measuring oxidative stress levels [33]. MDA levels increases under oxidative stress [34]. LDH is an intracellular enzyme present in cell cytoplasm, and its leakage from the cells is considered as an important indicator of cellular damage. In this study, treatment with HG-HUVEC-Exos increased the levels of MDA and LDH in VSMCs, as compared with NG-HUVEC-Exos group. The activity of SOD, an important antioxidant enzyme involved in antioxidant defenses, decreased significantly in VSMCs incubating with HG-HUVEC-Exos when compared with NG-HUVEC-Exos. Taken together, these results indicate that mitochondria played a critical role in inducing oxidative stress and subsequent cellular calcification/senescence of VSMCs induced by HG-HUVEC-Exos.

Exosomes are defined as microvesicles of 40–100 nm in diameter. The size distribution (74.6 ± 8.3 nm) and the enrichment of CD63 in isolated vesicles concur with the criteria for defining exosomes [35]. Because the role of exosomes is to transport miRNA, mRNA and protein from secretory cells to recipient cells [36], we conducted HPLC–MS/MS analysis and identified a total of 569 distinct proteins. The proteomic analysis assisted us to focus on VCAN among 179 proteins with altered levels. VCAN is a chondroitin sulphate proteoglycan that is found in the extracellular matrix (ECM) of many tissues in the body. A number of studies have shown that VCAN is associated with cardiovascular diseases including atherosclerosis, postangioplasty restenosis, pulmonary arterial hypertension and others [37–40]. VCAN is known to regulate smooth muscle cell retention of lipoproteins [40], growth and migration [41] and inflammation [42]. VCAN regulate cell adhesion, cell migration, and ECM assembly by direct or indirect interactions with cells and molecules [40]. Because of the overwhelming presence of VCAN in the ECM, there are only a few studies that have reported an intracellular localization of VCAN in different cells [43–45]. In this study, we provided clear evidence of VCAN’s presence in the mitochondria of VSMCs. Mitochondria usually act as metabolic headquarters. Molecular fuel, adenosine 5′-triphosphate (ATP) and ROS are produced in the mitochondria. Given the importance of mitochondria on cellular function, it is plausible that VCAN serves a vital role inside VSMCs. Indeed, our study suggests a function for VCAN in regulating mitochondrial function of VSMCs. Downregulation of VCAN by siRNA in HUVECs resulted in decreased oxidative stress levels and improved mitochondrial function in VSMCs. Collectively, these findings suggest that VCAN could, at least in part, induce mitochondrial dysfunction in VSMCs. These observations open a new avenue for study of VCAN, implying even more versatile roles for this proteoglycan than previously described.

Given the importance of VCAN to the normal function of ECM, it is difficult to differentiate between intracellular and extracellular functions of this proteoglycan. To this note, one of the limitations of this study is that we cannot rule out the possibility that the changes seen in VSMCs induced by HG-HUVEC-Exos were in fact secondary to altered VCAN in the extracellular environment. In addition, we cannot exclude the possibility that other exosomal proteins with altered levels might play a minor role in HG-HUVEC-Exos-induced VSMCs dysfunction, which needs future examination.

Despite the limitations, our data suggest a functional role for VCAN inside VSMCs. VCAN carried by HG-HUVEC-Exos promotes VSMCs calcification/senescence, probably by inducing mitochondrial dysfunction. Since VSMCs calcification/senescence could induce vascular dysfunction, blockage of the exosome-mediated transfer of VCAN between these two cells may serve as a potential therapeutic target against diabetic vascular complications. More work will be needed to explore this possibility and to better understand the intracellular roles of VCAN.

## Conclusion

In summary, here we report a study revealing that HG-HUVEC-Exos contain functional VCAN that is deliverable to VSMCs to induce calcification/senescence via modulation of mitochondrial function. A better understanding of the relationship between VCAN and mitochondrial dysfunction will help us to gain insight into the pathogenesis of diabetic vascular damage.

## Additional file

**Additional file 1: Figure S1.** The original film of Fig. 8a. HUVECs were transfected with negative control siRNA (con-siRNA) or VCAN siRNA (VCAN-siRNA) for 6 h. VCAN protein expression was assessed by Western blot. HUVECs (left panel), con-siRNA (middle panel) and VCAN-siRNA (right panel).

## Abbreviations

ECs: endothelial cells
VSMCs: vascular smooth muscle cells
ROS: reactive oxygen species
ALP: alkaline phosphatase
Runx2: Runt-related transcription factor
HUVECs: human umbilical vein endothelial cells
HUVEC-Exos: exosomes from human umbilical vein endothelial cells
NG/HG-HUVEC-Exos: exosomes from normal/high glucose stimulated HUVECs
NG: normal glucose
HG: high glucose
(SA-β-gal) staining: senescence-associated β-galactosidase staining
MDA: malondialdehyde
LDH: lactate dehydrogenase
SOD: superoxide dismutase
HPLC–MS/MS: high-performance liquid chromatography tandem mass spectrometry
GO analysis: Gene Ontology analysis
HADHA: hydroxyacyl-CoA dehydrogenase/3-ketoacyl-CoA thiolase/enoyl-CoA hydratase, alpha subunit
Cox-4: cytochrome oxidase-4
siRNA: small interfering RNA
VCAN: versican
ANOVA: one-way analysis of variance
ECM: extracellular matrix
HG-HUVEC-sup: supernatant isolated from HG stimulated HUVECs
con-siRNA: negative control siRNA
VCAN-siRNA: VCAN siRNA
