# Supplementary material for: Exosomes from hyperglycemia-stimulated vascular endothelial cells contain versican that regulate calcification/senescence in vascular smooth muscle cells
Source: Cell Biosci. 2019 Jan 3;9:1. doi: 10.1186/s13578-018-0263-x (PMC6317223; doi:10.1186/s13578-018-0263-x)
Supplement: Supplementary file 1 — Additional file 1: Figure S1. The original film of Fig. 8a. HUVECs were transfected with negative control siRNA (con-siRNA) or VCAN siRNA (VCAN-siRNA) for 6 h. VCAN protein expression was assessed by Western blot. HUVECs (left panel), con-siRNA (middle panel) and VCAN-siRNA (right panel). [file 13578_2018_263_MOESM1_ESM.docx]

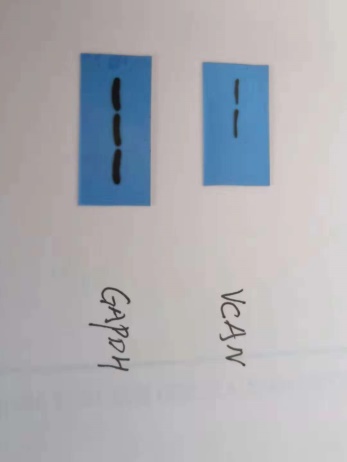


**Figure S1** The original film of Fig. 8A. HUVECs were transfected with negative control siRNA (con-siRNA) or VCAN siRNA (VCAN-siRNA) for 6 h. VCAN protein expression was assessed by Western blot. HUVECs (left panel), con-siRNA (middle panel) and VCAN-siRNA (right panel).
